# Synthesis and Thermotropic Studies of Two Novel Series of Kinked Liquid Crystals: 2-(4′-Alkoxybiphen-4-yl)-6-methylquinolines and 2-(6-Alkoxynaphthalen-2-yl)-6-methylquinolines

**DOI:** 10.3390/ijms15057579

**Published:** 2014-05-02

**Authors:** Win-Long Chia, Ker-Non Kuo, Shao-Hsun Lin

**Affiliations:** Department of Chemistry, Fu Jen Catholic University, New Taipei City 24205, Taiwan; E-Mails: sltjc0@gmail.com (K.-N.K.); brian730407@hotmail.com (S.-H.L.)

**Keywords:** liquid crystals, kinked structure, methylquinoline terminus, mesomorphic behavior

## Abstract

Two novel homologous series of kinked (Z-shaped) liquid crystalline compounds were synthesized using a short two-step reaction. Yields of 30%–40% and 51%–57% were obtained for 2-(4′-alkoxybiphen-4-yl)-6-methylquinolines (*n*O-PPQMe, *n* = 3–8) and 2-(6-alkoxynaphthalen-2-yl)-6-methylquinolines (*i*O-NpQMe, *i* = 3–7), respectively. Spectral analyses agreed with the expected structures. The thermotropic behaviors of these compounds were investigated using polarized optical microscopy and differential scanning calorimetry. An enantiotropic nematic phase appeared to be the main mesophase in these two series of kinked liquid crystalline compounds, and an additional enantiotropic smectic C phase appeared when *n* = 8.

## 1. Introduction

Although liquid crystal displays (LCDs) are ubiquitous in our daily life, the research of LCs is continuing to develop into many new scientific frontiers [1–3]. In the field of liquid crystals (LC), molecular structure is crucial for attaining the mesomorphic state. In the early twentieth century, Daniel Volander, a synthetic chemist, proposed that the crystalline-liquid (mesomorphic) state occurs when a molecular structure is as linear as possible [4]. Thus, liquid-crystal molecular structures are typically designed and constructed based on the principle that the shape anisotropy of a mesogen is rod-like. However, many aspects of the relationship between molecular structure and physical properties remain unknown, and the factors of a molecular structure favoring the formation of a nematic phase must be determined. Thus, the aim of this article is to illustrate one of the features in the molecular structure-property relationship of a nematic phase.

Because of the difficult synthesis of quinoline-containing liquid crystalline compounds, detailed comparisons of the trends in their homolog series have rarely been investigated. Quinoline-containing liquid crystalline compounds are typically synthesized using a cyclization reaction, such as the reaction of suitable substituted anilines and benzaldehydes with pyruvic acid, followed by decarboxylation of the corresponding carboxylic acids [5]. Other compounds are prepared by heating aniline with glycerin, 1,2-glycols, or unsaturated aldehydes, using the Skraup procedure or Doebner-Von Miller variation [6–11]. Another study reported the bisformylation of acetanilides, followed by cyclization with polyphosphoric acid, and subsequent conversion into chloroquinoline aldehydes, which are used as intermediates to further synthesize quinoline-containing liquid crystalline compounds [12–14]. Although these methods are extremely valuable for constructing crucial quinoline systems, most have a limited scope, involve numerous synthesis steps, and produce low yields. However, several recent patents have generated optical-switching elements with a high-speed response using quinoline-containing liquid crystalline compounds [15–17]. These reasons prompted us to seek a new method for synthesizing liquid crystalline compounds.

Previously, we reported a novel method for synthesizing quinoline-containing liquid crystalline compounds [18–20]. This paper reports the syntheses and thermotropic behaviors of two homologous series of 2-(4′-alkoxybiphen-4-yl)-6-methylquinolines (*n*O-PPQMe), in which *n* varies from 3–8 (propyl to octyl), and 2-(6-alkoxynaphthalen-2-yl)-6-methylquinolines (*i*O-NpQMe), in which *i* varies from 3–7 (propyl to heptyl). The liquid crystalline molecules designed in this study contained a linear mesogenic core with either one kink consisting of a quinoline moiety (as in *n*O-PPQMe) or two kinks consisting of naphthalene and quinoline moieties (as in *i*O-NpQMe).

## 2. Results and Discussion

### 2.1. Synthesis

**Scheme 1.**
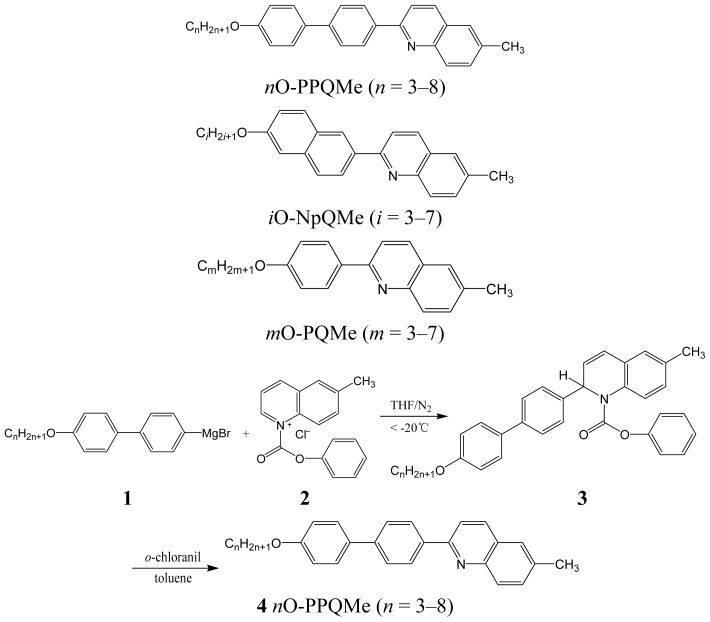
Synthetic route for the synthesis of *n*O-PPQMe.

Two homologous series of quinoline-containing liquid crystalline compounds, 2-(4′-alkoxy biphen-4-yl)-6-methylquinolines (*n*O-PPQMe, *n* = 3–8) and 2-(6-alkoxynaphthalen-2-yl)-6- methylquinolines (*i*O-NpQMe, *i* = 3–7), were obtained first through the regioselective addition of Grignard reagents to activated 1-acylquinolinium salts to preferentially form 1,2-dihydroquinolines, which were then aromatized through a mild oxidation reaction (Scheme 1).

Grignard reagents of **1** were prepared by reacting magnesium with appropriate 4′-alkoxy-4- bromobiphenylenes, which were obtained by reacting 4′-hydroxy-4-bromobiphenyl with the appropriate bromoalkanes. The reaction between 6-methylquinoline and phenyl chloroformate produced 6-methylquinolinium chlorides **2**. The reaction between Grignard **1** and **2** generated the expected 1,2-dihydroquinoline adduct **3**. Adducts **3** were then oxidized through treatment with *o*-chloranil to produce the desired 2-(4′-alkoxybiphen-4-yl)-6-methylquinolines **4** (*n*O-PPQMe, *n* = 3–8).

The proposed synthetic methodology favored Grignard α-regioselectivity at the quinoline ring. Because of the high polarity difference between the major α and minor γ products, trace amounts of γ products were easily separated using liquid chromatography with a methylene chloride: hexane eluant system at a 3:1 ratio. The yields of this two-step reaction ranged from 30% to 40%.

A similar procedure to that used for synthesizing *n*O-PPPQMe (*n* = 3–8) was used to generate *i*O-NpQMe (*i* = 3–7), using appropriate starting materials. Satisfactory yields (51%–57%) of 2-(6-alkoxynaphthalen-2-yl)-6-methylquinolines (*i*O-NpQMe, *i* = 3–7) were obtained. Highly pure products of *n*O-PPPQMe (*n* = 3–8) and *i*O-NpQMe (*i* = 3–7) were collected by recrystallizing them several times from toluene and ethyl acetate, respectively (Table 1).

### 2.2. Thermotropic Studies

The phase transition temperatures and associated enthalpy changes of the *n*O-PPQMe and *i*O-NpQMe compounds were determined using differential scanning calorimetry (DSC). The heating and cooling rates were set to 5 °C·min^−1^. The corresponding mesophases of the *n*O-PPQMe and *i*O-NpQMe compounds were identified by observing their textures using polarized optical microscopy (POM) as shown in Table 2.

All *n*O-PPQMe (*n* = 3–8) compounds exhibited an enantiotropic nematic phase. The longest alkoxy homolog (8O-PPQMe) exhibited an additional enantiotropic smectic C phase. The enantiotropic nematic phase appeared at a high temperature range of 188.7–311 °C. Because of the symmetry and poly-aromatic nature of *n*O-PPQMe, low solubility of these compounds in chloroform prohibited us from obtaining their ^13^C-NMR spectra. The nematic phase ranges of the *n*O-PPQMe (*n* = 3–8) compounds were wide. The widest nematic range was 107.5 °C of 3O-PPQMe during cooling. The nematic-to-isotropic transition temperature (*T*_NI_) of 3O-PPQMe extended even above its decomposition temperature, indicating that the series of *n*O-PPQMe compounds had high thermal stability.

**Figure 1.**
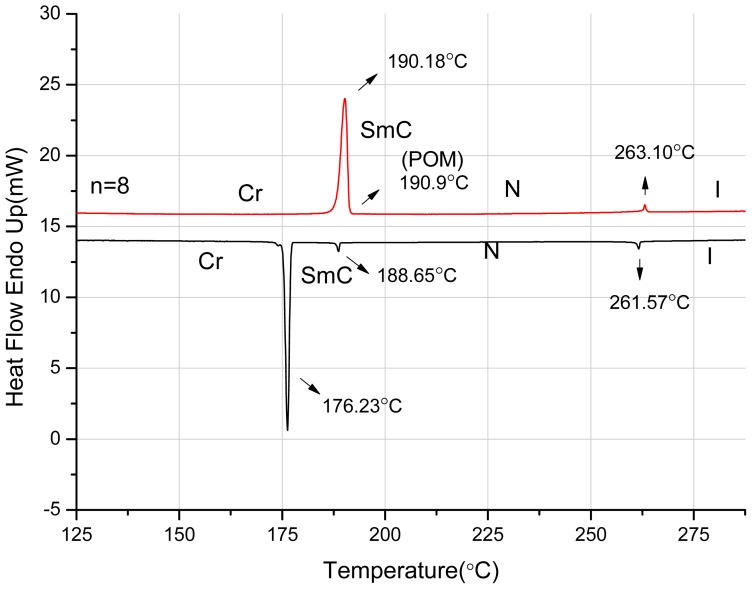
Thermograms of 8O-PPQMe obtained using a second DSC scan under a heating (red) and first cooling (black) rate of 5 °C·min^−1^; Cr = crystal phases; SmC = smectic C phase; N = nematic phase; I = isotropic phase.

**Figure 2.**
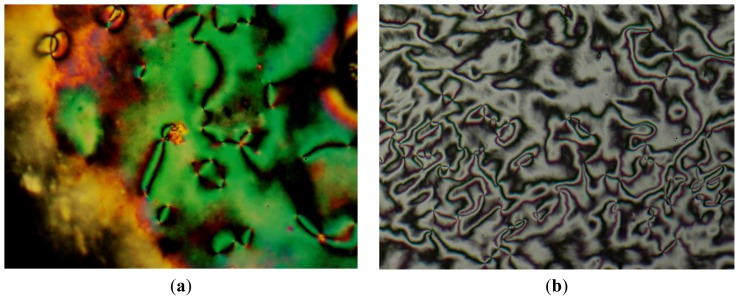
Polarized optical micrographs of 8O-PPQMe arise from isotropic phase on (**a**) heating to 260 °C; nematic Schlieren texture, ×200; and (**b**) cooling to 186 °C, smectic C texture, ×200.

A representative DSC thermogram of 8O-PPQMe is displayed in Figure 1. During the first cooling process, three exothermic peaks were observed at 261.6, 188.7, and 176.2 °C, whereas two endothermic peaks were observed at 190.2 and 263.1 °C during the second heating process. This phase transition behavior was also observed during the second cooling and third heating processes. During heating of 8O-PPQMe, a smectic C phase was observed within a narrow temperature range (less than 1 °C), using POM. The smectic C-to-nematic phase transition temperature (190.9 °C) was determined through a linear interpolation of the data from DSC and POM. Figure 2a,b display the nematic Schlieren texture and the smectic C phase of 8O-PPQMe, respectively.

**Figure 3.**
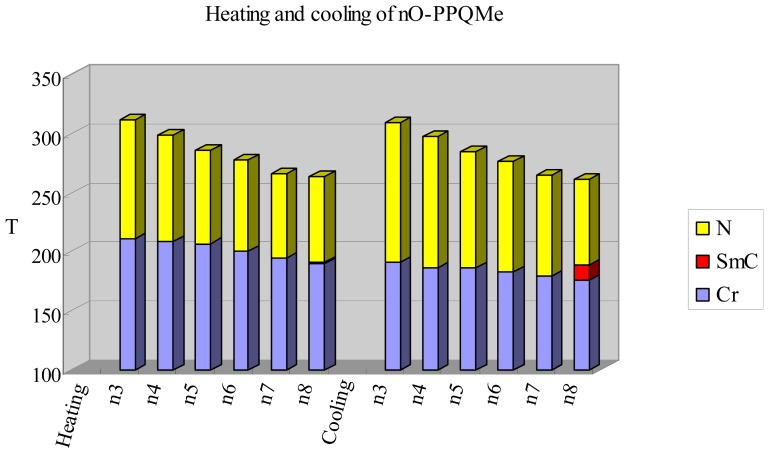
Plot of transition temperatures of heating and cooling cycle for compounds of 2-(4′-alkoxybiphen-4-yl)-6-methylquinoline (*n*O-PPQMe, *n* = 3–8).

Figure 3 displays the plot of transition temperatures of the heating and cooling cycle *versus* the number of carbon atoms in the terminal alkoxy chain of the *n*O-PPQMe homologs. The nematic-to-isotropic transition temperature (*T*_NI_) declined monotonically from 311 °C (estimated from POM) to 263.1 °C as the chain lengthened. If a line connects those of *T*_NI_ values, the line from those chains with an even number carbons were found only slightly higher than the line from odd number of carbons [21]. With increasing alkoxy chain length, a progressive decrease in the anisotropies of molecular polarizability was observed. The observed damping in the decrease of *T*_NI_ can be accounted for by the statistical increase in the numbers of possible non-extended conformations.

The wide nematic phase ranges observed during heating (71.9–100.4 °C) and cooling (72.9–107.5 °C) were associated with the high aspect ratio of the mesogenic core in the series of *n*O-PPQMe compounds. In *n*O-PPQMe compounds with one kink (quinoline) in the mesogenic core, the nematic phase was mainly the mesophase. Polymesomorphism did not appear in the *n*O-PPQMe series, except for a smectic C phase appearing in 8O-PPQMe. The asymmetrical, kinked, linear core structure also affected the melting and freezing processes. Thus, the melting and freezing transition temperatures occurred in a narrow range of 190.2–210.6 and 176.2–191.5 °C, respectively, and decreased slightly (within these temperature ranges) as the chain lengthened. Apparently, the kinked 6-methylquinoline terminus in *n*O-PPQMe compounds not only maintained the appearance of a nematic phase, but also affected the melting and freezing processes [22].

**Figure 4.**
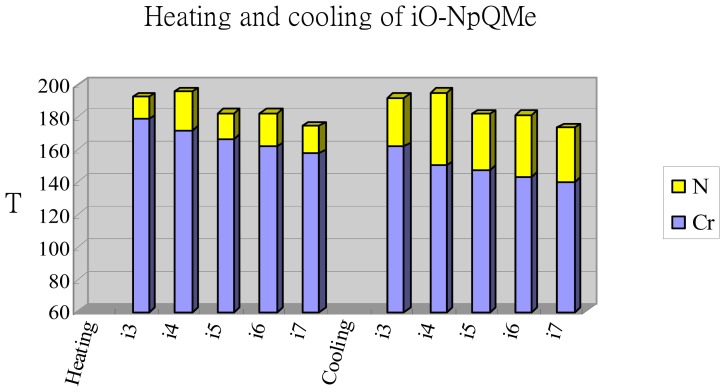
Plot of transition temperatures of heating and cooling cycle for compounds of 2-(6-alkoxynaphthalen-2-yl)-6-methylquinolines (*i*O-NpQMe, *i* = 3–7).

Figure 4 displays the plot of transition temperatures of the heating and cooling cycle *versus* the number of carbon atoms in the terminal alkoxy chain of the *i*O-NpQMe (*i* = 3–7) homologs. With two kinks (naphthalene, quinoline) along the molecular long axis, all *i*O-NpQMe (*i* = 3–7) compounds exhibited an enantiotropic nematic phase, and the enantiotropic nematic phase appeared at the medium–high temperature range of 140.2–192.2 °C.

By contrast to those of *n*O-PPQMe (*n* = 3–8) compounds, the nematic phase ranges of the *i*O-NpQMe (*i* = 3–7) compounds were considerably narrow, being 13.7–24.1 °C during heating and 29.7–44.6 °C during cooling. Although the cores of the *i*O-NpQMe compounds were only slightly shorter than those of the *n*O-PPQMe compounds, the cores of the *i*O-NpQMe compounds were wider than those of the *n*O-PPQMe compounds. The two kinks in the *i*O-NpQMe compounds were assumed to align in a transconformation in the nematic phase. Both adverse factors contributed to the aspect ratio of the *i*O-NpQMe compounds, and resulted in rounded molecules. Thus, the nematic phase ranges of the *i*O-NpQMe compounds were much narrower than those of the *n*O-PPQMe compounds.

The odd-even effect of the *T*_NI_(*T*_IN_) values was observed at the beginning of the *i*O-NpQMe series, in which *i* = 3, 4, 5 exhibited *T*_NI_(*T*_IN_) values 192.1 (191.4), 195.6 (194.9), and 182.3 (181.5) °C, respectively. By contrast, the *T*_NI_(*T*_IN_) values decreased as the chain lengthened when *i* > 5, in which *i* = 6, 7 are 182.0 (181.1), 174.0 (173.2) °C, respectively (see Table 2). The two-kinked core structures of the *i*O-NpQMe compounds also affected their melting and freezing processes. Thus, the melting and freezing transition temperatures occurred in a narrow range of 157.6–178.4 and 140.2–161.7 °C, respectively, and decreased monotonically (within these temperature ranges) as the chain lengthened.

**Figure 5.**
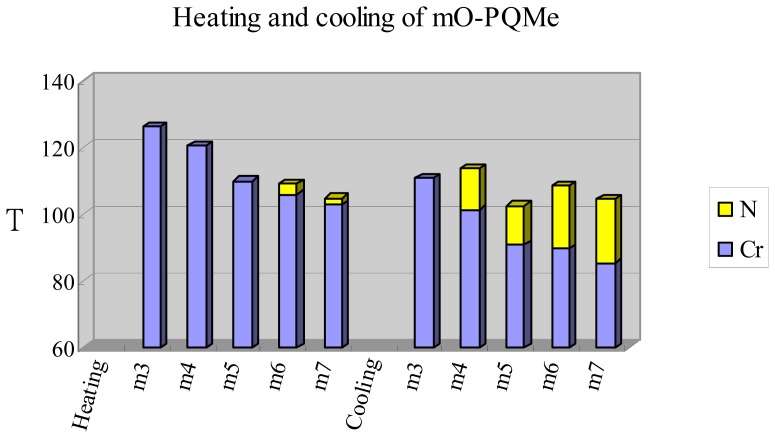
Plot of transition temperatures of heating and cooling cycle for compounds of 2-(4′-alkoxyphenyl)-6-methylquinoline (*m*O-PQMe, *m* = 3–7).

Figure 5 displays the plot of the transition temperatures of the heating and cooling cycle *versus* the number of carbon atoms in the terminal alkoxy chain of the *m*O-PQMe (*m* = 3–7) homologs [18]. With one kink (quinoline) present in the *m*O-PQMe mesogenic core, the nematic phase was still the sole mesophase. However, when the *m*O-PQMe compounds contained one phenyl group less than the *n*O-PPQMe and *i*O-NpQMe compounds did, the aspect ratios of their cores became too short to exhibit mesogenicity.

No mesophase was observed when *m* = 3, a monotropic nematic phase was observed when *m* = 4, 5, and an enantiotropic nematic phase was observed when *m* = 6, 7. Apparently, a stable nematic mesophase appeared gradually as the alkoxy chain lengthened. In other words, lengthening the alkoxy chain length of the *m*O-PQMe compounds not only enhanced their molecular aspect ratio, but also reduced their molecular melting point, thus revealing their mesogenic nature. Furthermore, the nematic phase of the series of *m*O-PQMe compounds appeared when the temperature dropped to the medium-low range of 80–110 °C. In addition, the nematic phase ranges of the *m*O-PQMe (*m* = 3–7) compounds were considerably narrow, and exhibited values of 3.3 and 2.0 °C, for *m* = 6, 7 during heating, and 12.4, 11.5, 19.1, and 19.3 °C, for *m* = 4, 5, 6, 7 during cooling, respectively.

The odd-even effect of the *T*_NI_(*T*_IN_) values can be clearly identified in the series of *m*O-PQMe compounds with a short mesogenic core (thus, low aspect ratio), in which the *T*_NI_(*T*_IN_) values for *m* = 4, 5, 6, 7 were – (113.7), – (102.5), 109.3 (108.7), and 104.9 (104.6) °C, respectively. The melting and freezing transition temperatures were observed occurring in a narrow range of 102.9–126.2 and 85.3–111.0 °C, respectively, and decreased (within this temperature range) as the chain lengthened.

## 3. Experimental Section

### 3.1. General

The chemical structures of the compounds were analyzed by ^1^H- and ^13^C-NMR spectra using a Bruker AC 300 spectrometer (Bruker Corporation, Billerica, MA, USA). Infrared (IR) spectra were carried out on a Perkin-Elmer 1600 Series spectrometer (Perkin-Elmer, Norwalk, CT, USA). The purity of the compounds was checked by thin-layer chromatography and further confirmed by elemental analysis.

Mesophases were chiefly identified by examination of the microscopic texture of samples sandwiched between two glass plates under a polarising optical microscope (POM; Olympus BH-2, Two Corporate Center Drive, Melville, NY, USA) equipped with a Mettler FP90/FP82HT hot stage (Mettler, Columbus, OH, USA). Phase transition temperatures and their corresponding transition enthalpies were determined by differential scanning calorimetry (DSC) using a Perkin-Elmer DSC 7 calorimeter at a scanning rate of 5 °C·min^−1^ (Perkin-Elmer, Norwalk, CT, USA). The program consists of first heating and followed by repeated cooling and heating cycles. Data from the repeated cycles were found to be identical to those of the first cooling and second heating. Therefore, only those data from the first cooling and second heating cycles are reported.

### 3.2. Synthesis

The starting material compounds, 6-methylquinoline, 6-bromo-2-naphthalenol and 4-bromo-4′-hydroxybiphenyl were purchased from Aldrich Chemical Co. (St. Louis, MO, USA) and used as-received. Phenyl chloroformate and *n*-bromoalkanes were distilled under an inert nitrogen atmosphere immediately before use. Anhydrous organic solvents, toluene and tetrahydrofuran (THF), were first heated at reflux over sodium and then distilled under nitrogen before use. Column chromatography was carried out with silica gel (MN Kieselgel 60, 70–230 mesh; Duren, Germany). The purity of the compounds was checked by thin-layer chromatography and further confirmed by elemental analysis.

#### 3.2.1. Syntheses of 2-(4′-Alkoxybiphen-4-yl)-6-methylquinolines (*n*O-PPQMe, *n* = 3–8)

The synthesis of 2-(4′-alkoxybiphen-4-yl)-6-methylquinolines was carried out according to the synthetic methods outlined in Scheme 1. The entire synthetic procedures were completed in a short two-step process with overall yields in the range of 30%–40% (Table 1). For 3O-PPQMe in **3**: To a (Grignard) solution of 4-bromo-4′-ethoxybiphenyl (10 mmol) in THF (20 mL) was added freshly dried magnesium granules (11 mmol) under an inert nitrogen atmosphere. The Grignard solution **1** was then slowly added by syringe into a preformed solution of 6-methylquinolinium chloride **2**, which was prepared from phenyl chloroformate (10 mmol) and 3-methylquinoline (10 mmol) in dry THF (20 mL) under −20 °C for 30 min. The resulting solution was heated slowly to room temperature and stirred for another 8 h. After evaporating the THF, the residue was extracted with Et_2_O. The organic layer was further washed once with 20% NH_4_Cl solution and twice with distilled water and brine and dried with magnesium sulfate. For 3O-PPQMe in **4**: To a solution of dry toluene (20 mL) and compound **3** (10 mmol) was added about 1.5 eq. *o*-chloranil. The reaction mixture was heated to reflux for about 3 h under inert nitrogen atmosphere and then quenched by adding 1 N NaOH (25 mL) and Et_2_O (25 mL) and filtered through Celite (Duren, Germany). Normal aqueous work-up and isolation with column chromatography (methylene chloride:hexane = 3:1) affords an overall two-step reaction yield of 2-(4-propoxybiphen-4-yl)-6-methylquinoline **4** (3O-PPQMe) (37%). The crude product of 3O-PPQMe was further purified by re-crystallization several times from a mixed solvent of methylene chloride and ethyl acetate. The other *n*O-PPQMe homologues were synthesized essentially by the same procedure as described above for the *n* = 3 homologue. All compounds gave satisfactory ^1^H-NMR, IR and elemental analysis as illustrated below. ^13^C-NMR of these linear poly-aromatic *n*O-PPQMe compounds was not obtained due to their low solubility in CDCl_3_.

##### 3.2.1.1. 2-(4′-Propoxybiphen-4-yl)-6-methylquinoline (3O-PPQMe)

^1^H-NMR (CDCl_3_): δ 8.21 (d, 2H, *J* = 8.4 Hz, center-benzene), 8.14 (d, 1H, *J* = 8.7 Hz, quinoline), 8.08 (d, 1H, *J* = 8.4 Hz, quinoline), 7.88 (d, 1H, *J* = 8.7 Hz, quinoline), 7.72 (d, 2H, *J* = 8.4 Hz, center-benzene), 7.55–7.62 (m, 4H, 2 in outer-benzene, 2 in quinoline), 7.00 (d, 2H, *J* = 9.0 Hz, outer-benzene), 3.99 (t, 2H, *J* = 6.6 Hz, –OCH_2_), 2.56 (s, 3H, –CH_3_), 1.84 (ses, 2H, *J* = 7.2 Hz, –CH_2_), 1.07 (t, 3H, *J* = 7.5 Hz, –CH_3_). IR (ATR): cm^−1^ 3039 (aromatic C–H stretch), 2964 (aliphatic C–H asymmetric stretch), 2876 (aliphatic C–H symmetric stretch), 1595 (ring stretch), 1491 (ring stretch), 1205 (asymmetric C–O–C stretch), 1069 (symmetric C–O–C stretch), 823 (out-of-plane C–H bend). HRFAB mass obsd *m*/*z* 353.1772; mass calcd for C_25_H_23_NO 353.4600. Anal. Calcd for C_25_H_23_NO: C, 84.95; H, 6.56; N, 3.96. Found: C, 84.52; H, 6.56; N, 3.96.

##### 3.2.1.2. 2-(4′-Butoxybiphen-4-yl)-6-methylquinoline (4O-PPQMe)

^1^H-NMR (CDCl_3_): δ 8.21 (d, 2H, *J* = 8.4 Hz, center-benzene), 8.14 (d, 1H, *J* = 8.4 Hz, quinoline), 8.06 (d, 1H, *J* = 8.4 Hz, quinoline), 7.88 (d, 1H, *J* = 8.7 Hz, quinoline), 7.71 (d, 2H, *J* = 8.4 Hz, center-benzene), 7.55–7.62 (m, 4H, 2 in outer-benzene, 2 in quinoline), 7.00 (d, 2H, *J* = 8.7 Hz, outer-benzene), 4.03 (t, 2H, *J* = 6.6 Hz, –OCH_2_), 2.56 (s, 3H, –CH_3_), 1.82 (quin, 2H, *J* = 6.6 Hz, –CH_2_), 1.51 (ses, 2H, *J* = 7.2 Hz, –CH_2_), 1.00 (t, 3H, *J* = 7.5 Hz, –CH_3_). IR (ATR): cm^−1^ 3042 (aromatic C–H stretch), 2957 (aliphatic C–H asymmetric stretch), 2872 (aliphatic C–H symmetric stretch), 1595 (ring stretch), 1492 (ring stretch), 1206 (asymmetric C–O–C stretch), 1040 (symmetric C–O–C stretch), 824 (out-of-plane C–H bend). HRFAB mass obsd *m*/*z* 367.1931; mass calcd for C_26_H_25_NO 367.4800. Anal. Calcd for C_26_H_25_NO: C, 84.98; H, 6.86; N, 3.81. Found: C, 84.82; H, 7.01; N, 3.80.

##### 3.2.1.3. 2-(4′-Pentoxybiphen-4-yl)-6-methylquinoline (5O-PPQMe)

^1^H-NMR (CDCl_3_): δ 8.21 (d, 2H, *J* = 8.1 Hz, center-benzene), 8.14 (d, 1H, *J* = 8.4 Hz, quinoline), 8.07 (d, 1H, *J* = 8.4 Hz, quinoline), 7.88 (d, 1H, *J* = 8.7 Hz, quinoline), 7.72 (d, 2H, *J* = 8.1 Hz, center-benzene), 7.55–7.62 (m, 4H, 2 in outer-benzene, 2 in quinoline), 7.00 (d, 2H, *J* = 8.4 Hz, outer-benzene), 4.02 (t, 2H, *J* = 6.6 Hz, –OCH_2_), 2.56 (s, 3H, –CH_3_), 1.83 (quin, 2H, *J* = 6.9 Hz, –CH_2_), 1.35–1.53 (m, 4H, –CH_2_), 0.95 (t, 3H, *J* = 7.2 Hz, –CH_3_). IR (ATR): cm^−1^ 3031 (aromatic C–H stretch), 2936 (aliphatic C–H asymmetric stretch), 2866 (aliphatic C–H symmetric stretch), 1596 (ring stretch), 1492 (ring stretch), 1207 (asymmetric C–O–C stretch), 1029 (symmetric C–O–C stretch), 824 (out-of-plane C–H bend). HRFAB mass obsd *m*/*z* 381.2088; mass calcd for C_27_H_27_NO 381.5100. Anal. Calcd for C_27_H_27_NO: C, 85.00; H, 7.13; N, 3.67. Found: C, 84.70; H, 7.21; N, 3.52.

##### 3.2.1.4. 2-(4′-Hexoxybiphen-4-yl)-6-methylquinoline (6O-PPQMe)

^1^H-NMR (CDCl_3_): δ 8.25 (d, 2H, *J* = 8.4 Hz, center-benzene), 8.17 (d, 1H, *J* = 8.4 Hz, quinoline), 8.03 (d, 1H, *J* = 8.4 Hz, quinoline), 7.92 (d, 1H, *J* = 8.7 Hz, quinoline), 7.73 (dd, 2H, *J* = 8.4 Hz, center-benzene), 7.56–7.66 (m, 4H, 2 in outer-benzene, 2 in quinoline), 7.00 (d, 2H, *J* = 9.0 Hz, outer-benzene), 4.01 (t, 2H, *J* = 6.6 Hz, –OCH_2_), 2.55 (s, 3H, –CH_3_), 1.81 (quin, 2H, *J* = 6.9 Hz, –CH_2_), 1.30–1.56 (m, 6H, –CH_2_), 0.92 (t, 3H, *J* = 7.2 Hz, –CH_3_). IR (ATR): cm^−1^ 3041 (aromatic C–H stretch), 2954 (aliphatic C–H asymmetric stretch), 2865 (aliphatic C–H symmetric stretch), 1595 (ring stretch), 1492 (ring stretch), 1206 (asymmetric C–O–C stretch), 1031 (symmetric C–O–C stretch), 824 (out-of-plane C–H bend). HRFAB mass obsd *m*/*z* 395.2252; mass calcd for C_28_H_29_NO 395.5400. Anal. Calcd for C_28_H_29_NO: C, 85.02; H, 7.39; N, 3.54. Found: C, 85.20; H, 7.57; N, 3.52.

##### 3.2.1.5. 2-(4′-Heptoxybiphen-4-yl)-6-methylquinoline (7O-PPQMe)

^1^H-NMR (CDCl_3_): δ 8.21 (d, 2H, *J* = 8.4 Hz, center-benzene), 8.14 (d, 1H, *J* = 8.7 Hz, quinoline), 8.07 (d, 1H, *J* = 8.4 Hz, quinoline), 7.88 (d, 1H, *J* = 8.7 Hz, quinoline), 7.71 (d, 2H, *J* = 8.4 Hz, center-benzene), 7.55–7.62 (m, 4H, 2 in outer-benzene, 2 in quinoline), 7.00 (d, 2H, *J* = 8.7 Hz, outer-benzene), 4.01 (t, 2H, *J* = 6.6 Hz, –OCH_2_), 2.56 (s, 3H, –CH_3_), 1.82 (quin, 2H, *J* = 6.9 Hz, –CH_2_), 1.29–1.53 (m, 8H, –CH_2_), 0.90 (t, 3H, *J* = 6.9 Hz, –CH_3_). IR (ATR): cm^−1^ 3041 (aromatic C–H stretch), 2953 (aliphatic C–H asymmetric stretch), 2860 (aliphatic C–H symmetric stretch), 1595 (ring stretch), 1492 (ring stretch), 1207 (asymmetric C–O–C stretch), 1026 (symmetric C–O–C stretch), 824 (out-of-plane C–H bend). HRFAB mass obsd *m*/*z* 409.2406; mass calcd for C_29_H_31_NO 409.5600. Anal. Calcd for C_29_H_31_NO: C, 84.82; H, 7.57; N, 3.41. Found: C, 85.04; H, 7.63; N, 3.42.

##### 3.2.1.6. 2-(4′-Octoxybiphen-4-yl)-6-methylquinoline (8O-PPQMe)

^1^H-NMR (CDCl_3_): δ 8.21 (d, 2H, *J* = 7.8 Hz, center-benzene), 8.14 (d, 1H, *J* = 8.7 Hz, quinoline), 8.07 (d, 1H, *J* = 8.4 Hz, quinoline), 7.88 (d, 1H, *J* = 8.7 Hz, quinoline), 7.72 (d, 2H, *J* = 8.4 Hz, center-benzene), 7.55–7.62 (m, 4H, 2 in outer-benzene, 2 in quinoline), 7.00 (d, 2H, *J* = 8.1 Hz, outer-benzene), 4.01 (t, 2H, *J* = 6.6 Hz, –OCH_2_), 2.56 (s, 3H, –CH_3_), 1.82 (quin, 2H, *J* = 6.9 Hz, –CH_2_), 1.25–1.53 (m, 10H, –CH_2_), 0.89 (t, 3H, *J* = 6.6 Hz, –CH_3_). IR (ATR): cm^−1^ 3042 (aromatic C–H stretch), 2956 (aliphatic C–H asymmetric stretch), 2854 (aliphatic C–H symmetric stretch), 1596 (ring stretch), 1492 (ring stretch), 1206 (asymmetric C–O–C stretch), 1030 (symmetric C–O–C stretch), 824 (out-of-plane C–H bend). HRFAB mass obsd *m*/*z* 423.2569; mass calcd for C_30_H_33_NO 423.5900. Anal. Calcd for C_30_H_33_NO: C, 85.06; H, 7.85; N, 3.31. Found: C, 84.60; H, 7.94; N, 3.25.

#### 3.2.2. 2-(6-Alkoxynaphthalen-2-yl)-6-methylquinolines (*i*O-NpQMe, *i* = 3–7)

##### 3.2.2.1. 2-(6-Propyloxynaphthalen-2-yl)-6-methylquinolines (3O-NpQMe)

^1^H-NMR (CDCl_3_): δ 8.53 (d, 1H, *J* = 1.5 Hz, naphthalene), 8.32 (dd, 1H, *J_1_* = 8.7 Hz, *J_2_* = 1.8 Hz, naphthalene), 8.09–8.14 (m, 2H, quinoline), 7.95 (d, 1H, *J* = 8.7 Hz, quinoline), 7.84–7.90 (m, 2H, naphthalene), 7.54–7.60 (m, 2H, quinoline), 7.16–7.23 (m, 2H, naphthalene), 4.07 (t, 2H, *J* = 6.6 Hz, –OCH_2_), 2.56 (s, 3H, –CH_3_), 1.89 (ses, 2H, *J* = 7.2 Hz, –CH_2_), 1.11 (t, 3H, *J* = 7.5 Hz, –CH_3_). ^13^C-NMR (CDCl_3_): ppm 157.9, 156.5, 147.0, 136.0, 136.0, 135.2, 134.9, 131.9, 130.3, 129.4, 129.0, 127.3, 127.2, 126.8, 126.4, 125.5, 119.5, 119.0, 106.5, 69.6, 22.6, 21.6, 10.6. IR (KBr): cm^−1^ 3053 (aromatic C–H stretch), 2962 (aliphatic C–H asymmetric stretch), 2876 (aliphatic C–H symmetric stretch), 1593 (ring stretch), 1489 (ring stretch), 1173 (asymmetric C–O–C stretch), 1062 (symmetric C–O–C stretch), 822 (out-of-plane C–H bend). Anal. Calcd for C_23_H_21_NO: C, 84.37; H, 6.46; N, 4.28. Found: C, 84.01; H, 6.45; N, 4.16.

##### 3.2.2.2. 2-(6-Butyloxynaphthalen-2-yl)-6-methylquinolines (4O-NpQMe)

^1^H-NMR (CDCl_3_): δ 8.53 (s, 1H, naphthalene), 8.31 (dd, 1H, *J*_1_ = 8.7 Hz, *J_2_* = 1.8 Hz, naphthalene), 8.08–8.18 (m, 2H, quinoline), 7.97 (d, 1H, *J* = 8.7 Hz, quinoline), 7.83–7.90 (m, 2H, naphthalene) 7.55–7.62 (m, 2H, quinoline), 7.16–7.22 (m, 2H, naphthalene), 4.12 (t, 2H, *J* = 6.6 Hz, –OCH_2_), 2.56 (s, 3H, –CH_3_), 1.86 (quin, 2H, *J* = 6.9 Hz, –CH_2_), 1.56 (ses, 2H, *J* = 7.5 Hz, –CH_2_), 1.02 (t, 3H, *J* = 7.5 Hz, –CH_3_). ^13^C-NMR (CDCl_3_): ppm 158.1, 156.3, 146.3, 136.7, 136.3, 135.3, 134.1, 132.3, 130.4, 128.9, 128.9, 127.4, 127.2, 126.4, 126.4, 125.5, 119.6, 119.1, 106.4, 67.8, 31.3, 21.6, 19.4, 13.9. IR (KBr): cm^−1^ 3008 (aromatic C–H stretch), 2922 (aliphatic C–H asymmetric stretch), 2856 (aliphatic C–H symmetric stretch), 1596 (ring stretch), 1464 (ring stretch), 1206 (asymmetric C–O–C stretch), 1014 (symmetric C–O–C stretch), 822 (out-of-plane C–H bend). Anal. Calcd for C_24_H_23_NO: C, 84.42; H, 6.79; N, 4.10. Found: C, 84.36; H, 6.80; N, 4.08.

##### 3.2.2.3. 2-(6-Pentyloxynaphthalen-2-yl)-6-methylquinolines (5O-NpQMe)

^1^H-NMR (CDCl_3_): δ 8.53 (s, 1H, naphthalene), 8.31 (dd, 1H, *J*_1_ = 8.7 Hz, *J*_2_ = 1.8 Hz, naphthalene), 8.08–8.15 (m, 2H, quinoline), 7.95 (d, 1H, *J* = 8.7 Hz, quinoline), 7.83–7.90 (m, 2H, naphthalene), 7.55–7.60 (m, 2H, quinoline), 7.16–7.23 (m, 2H, naphthalene), 4.10 (t, 2H, *J* = 6.6 Hz, –OCH_2_), 2.56 (s, 3H, –CH_3_), 1.88 (quin, 2H, *J* = 7.5 Hz, –CH_2_), 1.38–1.58 (m, 4H, –CH_2_), 0.97 (t, 3H, *J* = 7.2 Hz, –CH_3_). ^13^C-NMR (CDCl_3_): ppm 157.9, 156.5, 147.0, 136.0, 136.0, 135.2, 134.9, 131.9, 130.3, 129.4, 129.0, 127.3, 127.2, 126.8, 126.4, 125.5, 119.5, 119.0, 106.5, 68.1, 29.0, 28.3, 22.5, 21.6, 14.1. IR (KBr): cm^−1^ 3008 (aromatic C–H stretch), 2957 (aliphatic C–H asymmetric stretch), 2856 (aliphatic C–H symmetric stretch), 1596 (ring stretch), 1489 (ring stretch), 1203 (asymmetric C–O–C stretch), 1047 (symmetric C–O–C stretch), 822 (out-of-plane C–H bend). Anal. Calcd for C_25_H_25_NO: C, 84.47; H, 7.09; N, 3.94. Found: C, 84.26; H, 7.10; N, 3.92.

##### 3.2.2.4. 2-(6-Hexyloxynaphthalen-2-yl)-6-methylquinolines (6O-NpQMe)

^1^H-NMR (CDCl_3_): δ 8.53 (s, 1H, naphthalene), 8.31 (dd, 1H, *J*_1_ = 8.7 Hz, *J*_2_ = 1.8 Hz, naphthalene), 8.07–8.18 (m, 2H, quinoline), 7.97 (d, 1H, *J* = 8.7 Hz, quinoline), 7.84–7.90 (m, 2H, naphthalene), 7.55–7.62 (m, 2H, quinoline), 7.16–7.22 (m, 2H, naphthalene), 4.11 (t, 2H, *J* = 6.6 Hz, –OCH_2_), 2.56 (s, 3H, –CH_3_), 1.87 (quin, 2H, *J* = 6.9 Hz, –CH_2_), 1.29–1.58 (m, 6H, –CH_2_), 0.93 (t, 3H, *J* = 6.9 Hz, –CH_3_). ^13^C-NMR (CDCl_3_): ppm 157.9, 156.5, 147.0, 136.0, 136.0, 135.2, 134.9, 131.9, 130.3, 129.4, 128.9, 127.3, 127.2, 126.7, 126.4, 125.5, 119.5, 118.9, 106.5, 68.1, 31.7, 29.3, 25.8, 22.7, 21.6, 14.1. IR (KBr): cm^−1^ 3008 (aromatic C–H stretch), 2922 (aliphatic C–H asymmetric stretch), 2856 (aliphatic C–H symmetric stretch), 1596 (ring stretch), 1489 (ring stretch), 1203 (asymmetric C–O–C stretch), 1042 (symmetric C–O–C stretch), 822 (out-of-plane C–H bend). Anal. Calcd for C_26_H_27_NO: C, 84.51; H, 7.37; N, 3.79. Found: C, 84.30; H, 7.34; N, 3.72.

##### 3.2.2.5. 2-(6-Heptyloxynaphthalen-2-yl)-6-methylquinolines (7O-NpQMe)

^1^H-NMR (CDCl_3_): δ 8.54 (s, 1H, naphthalene), 8.32 (dd, 1H, *J*_1_ = 8.7 Hz, *J*_2_ = 1.2 Hz, naphthalene), 8.08–8.16 (m, 2H, quinoline), 7.96 (dd, 1H, *J*_1_ = 8.7 Hz, *J*_2_ = 1.8 Hz, quinoline), 7.83–7.90 (m, 2H, naphthalene), 7.54–7.60 (m, 2H, quinoline), 7.16–7.22 (m, 2H, naphthalene), 4.10 (t, 2H, *J* = 6.6 Hz, –OCH_2_), 2.56 (s, 3H, –CH_3_), 1.88 (quin, 2H, *J* = 7.2 Hz, –CH_2_), 1.29–1.58 (m, 8H, –CH_2_), 0.93 (t, 3H, *J* = 6.9 Hz, –CH_3_). ^13^C-NMR (CDCl_3_): ppm 157.9, 156.5, 147.0, 136.0, 136.0, 135.2, 134.9, 131.9, 130.3, 129.4, 129.0, 127.3, 127.2, 126.8, 126.4, 125.5, 119.5, 119.0, 106.5, 68.1, 31.9, 29.3, 29.1, 26.1, 22.7, 21.6, 14.1. IR (KBr): cm^−1^ 3008 (aromatic C–H stretch), 2922 (aliphatic C–H asymmetric stretch), 2856 (aliphatic C–H symmetric stretch), 1596 (ring stretch), 1464 (ring stretch), 1206 (asymmetric C–O–C stretch), 1014 (symmetric C–O–C stretch), 822 (out-of-plane C–H bend). Anal. Calcd for C_27_H_29_NO: C, 84.55; H, 7.62; N, 3.65. Found: C, 84.60; H, 7.54; N, 3.60.

## 4. Conclusions

In conclusion, two novel homologous series of kinked (Z-shaped) liquid crystalline compounds, *n*O-PPQMe (*n* = 3–8) and *i*O-NpQMe (*i* = 3–7), were synthesized, and their thermotropic behaviors were examined. With one kink (quinoline) and high aspect ratio of the mesogenic core in the series of *n*O-PPQMe compounds, high *T*_NI_ values and wide nematic phase ranges were observed. With two kinks (naphthalene, quinoline) as in *i*O-NpQMe (*i* = 3–7) compounds, although enantiotropic nematic phases were maintained, considerably lower *T*_NI_(*T*_IN_) values and narrower nematic phase ranges resulted. With one kink (quinoline) but a short mesogenic core as in the series of *m*O-PQMe compounds, although the nematic phase was the sole mesophase, mesogenicity was barely observed. In all, a linear mesogen with one kinked structure and high aspect ratio favors the formation of nematic phase.

## Figures and Tables

**Table 1.** Yields of 2-(4′-alkoxybiphen-4-yl)-6-methylquinolines (*n*O-PPQMe, *n* = 3–8) and 2-(6-alkoxynaphthalen-2-yl)-6-methylquinolines (*i*O-NpQMe, *i* = 3–7).

Entry (*n*)	Alkyl group	Yield a (%)
*n* = 3	Propyl	37
*n* = 4	Butyl	35
*n* = 5	Pentyl	30
*n* = 6	Hexyl	39
*n* = 7	Heptyl	40
*n* = 8	Octyl	37
*i* = 3	Propyl	51
*i* = 4	Butyl	52
*i* = 5	Pentyl	52
*i* = 6	Hexyl	57
*i* = 7	Heptyl	55

a Isolated yields are quoted after recrystallization.

**Table 2.** Phase transition temperatures (°C) and corresponding transition enthalpies (kJ·mol^−1^), in parentheses, for homologous series of *n*O-PPPQMe, *n* = 3–8, and *i*O-NpQMe, *i* = 3–7.

Compound *n*O-PPQMe or *i*O-NpQMe	Phase transition temperatures (°C) and their corresponding transition enthalpies (kJ·mol^−1^)	

	Heating a	Cooling a

*n* = 3	Cr 210.6(26.3) N 311 b I	I 309 b N 191.5(22.3) Cr
*n* = 4	Cr 209.0(30.7) N 299.1(0.9) I	I 297.7(0.8) N 186.7(27.9) Cr
*n* = 5	Cr 206.9(35.8) N 286.0(0.8) I	I 284.4(0.8) N 186.6(33.7) Cr
*n* = 6	Cr 200.0(28.9) N 278.1(0.8) I	I 276.7(0.8) N 182.7(27.0) Cr
*n* = 7	Cr 194.5(27.7) N 266.4(0.4) I	I 264.4(0.5) N 179.4(26.6) Cr
*n* = 8	Cr 190.2(32.8) SmC 190.9 c N 263.1(0.8) I	I 261.6(1.0) N 188.7(0.9) SmC 176.2(30.4) Cr
*i* = 3	Cr 178.5(36.6) N 192.1(0.4) I	I 191.5(0.4) N 161.7(32.5) Cr
*i* = 4	Cr 171.5(32.5) N 195.6(0.4) I	I 194.9(0.5) N 150.3(31.2) Cr
*i* = 5	Cr 166.3(32.9) N 182.3(0.5) I	I 181.6(0.4) N 147.2(28.2) Cr
*i* = 6	Cr 161.7(31.9) N 182.0(0.5) I	I 181.2(0.5) N 143.1(29.8) Cr
*i* = 7	Cr 157.6(32.2) N 174.1(0.5) I	I 173.2(0.4) N 140.2(31.3) Cr

a Scan rate: 5 °C·min^−1^ for all samples; Cr = crystal phases; SmC = smectic C phase; N = nematic phase; I = isotropic phase;

b The temperatures were observed using POM because of the sample’s decomposition;

c The temperature was determined through linear interpolation of the data from DSC and POM; The peak of the SmC-to-N transition was buried in the peak of the Cr-to-SmC transition during heating, and they were separated during cooling.
